# Regulatory pattern of abnormal promoter CpG island methylation in the glioblastoma multiforme classification

**DOI:** 10.3389/fgene.2022.989985

**Published:** 2022-09-19

**Authors:** Rendong Wang, Lei Zhao, Shijia Wang, Xiaoxiao Zhao, Chuanyu Liang, Pei Wang, Dongguo Li

**Affiliations:** ^1^ School of Biomedical Engineering, Capital Medical University, Beijing, China; ^2^ Beijing Key Laboratory of Fundamental Research on Biomechanics in Clinical, Capital Medical University, Beijing, China; ^3^ Department of Anesthesiology, Xuanwu Hospital, Capital Medical University, Beijing, China

**Keywords:** glioblastoma 1, subtype classification 2, CpG island 3, DNA methylation 4, tumor microenviroment 5, single-cell RNA sequencing 6, intercellular communication 7

## Abstract

Glioblastoma (GBM) is characterized by extensive genetic and phenotypic heterogeneity. However, it remains unexplored primarily how CpG island methylation abnormalities in promoter mediate glioblastoma typing. First, we presented a multi-omics scale map between glioblastoma sample clusters constructed based on promoter CpG island (PCGI) methylation-driven genes, using datasets including methylation profiles, expression profiles, and single-cell sequencing data from multiple highly annotated public clinical cohorts. Second, we identified differences in the tumor microenvironment between the two glioblastoma sample clusters and resolved key signaling pathways between cell clusters at the single-cell level based on comprehensive comparative analyses to investigate the reasons for survival differences between two of these clusters. Finally, we developed a diagnostic map and a prediction model for glioblastoma, and compared theoretical differences of drug sensitivity between two glioblastoma sample clusters. In summary, this study established a classification system for dissecting promoter CpG island methylation heterogeneity in glioblastoma and provides a new perspective for the diagnosis and treatment of glioblastoma.

## Introduction

Glioblastoma (GBM) is a malignant primary brain cancer characterized by high infiltration into the parenchyma and wide phenotypic heterogeneity (Hua et al., 2015). Despite advances in surgical techniques and clinical regimens, the standard therapies, including surgical resection, chemotherapy, are predominantly ineffective for GBMs due to therapeutic resistance, rapid recurrence, and the patient outcomes remain between 12 and 15 months survival rate, 5-year survival rates at only 10% (Tao et al., 2020). In light of the molecular complexity and histopathological grading of GBM (Vitucci et al., 2017), there is a critical need to complement the inaccurate prediction of disease progression and the deviation of therapy with genomic information.

The significant factors contributing to the pathogenesis of GBM were epigenetic molecular mechanisms (Kosti et al., 2020). DNA methylation, the most common epigenetic event in cancer, contributes to carcinogenesis and frequently occurs in the promoter region of genes (Agundez et al., 2011; Wang et al., 2022). With the help of multi-omics datasets, profiles of GBM at the transcriptome and methylation levels have been increasingly reported to investigate the extensive heterogeneity in the tumor and single-cell level regarding transcriptomic expression (Oh et al., 2020). Several extensive cohort studies indicate an important association between DNA methylation of the promoter region and phenotypic of GBM (Guo et al., 2015). For instance, the discordance of promoter methylation with O-6-methylguanine-DNA-methyltransferase (MGMT) expression in GBM has been a plausible strategy for sensitizing temozolomide (TMZ) therapy and provides a strong rationale for the development of new drugs (Yi et al., 2019). Furthermore, numerous potential prognostic biomarkers, including long non-coding RNA (lncRNA) and mRNA, were identified with aberrant methylation (Han et al., 2020). The characterization of the epigenome by DNA methylation assay has been progressively used to stratify and integrate molecular and phenotypic features. Nevertheless, with advances in genomics, the single-gene methylation status has limited its clinical utility.

During cancer development, aberrant DNA methylation occurs within the gene promoter, CpG island, and their shores (Hardy et al., 2017). However, CpG island has received little individual attention. CpG sites methylation patterns are believed to differ considerably between GBM patients (Etcheverry et al., 2010). In particular, some cancers show an apparent CpG island methylator phenotype (CIMP), of which a critical milestone highlighting the clinical importance of the epigenetic profile of gliomas was the discovery of the glioma CpG island methylation phenotype (G-CIMP) (Northcott et al., 2017; Ogino et al., 2018). Specifically, patients carrying G-CIMP have a better prognosis than patients who do not carry this phenotype. The clusters identified by separating Isocitrate dehydrogenase (IDH) mutation status showed overall concordance with G-CIMP, which exemplifies the particularity of CpG island in the molecular diagnosis of GBM (Geisenberger et al., 2015; Park et al., 2019). Recent studies also suggest that the tumor microenvironment (TME) plays an essential role in clinical outcomes and response to therapy (Gangoso et al., 2021). The tumor microenvironment of GBM contains a large number of infiltrating macrophages (Chen et al., 2019). However, few studies have assessed the epigenetic alterations and the TME simultaneously, especially at the single-cell level. Here we explored a comprehensive genomic and transcriptomic analysis. We resolve the comprehensive characterization of GBM subgroups by integrating CpG island methylation, expression profiling, and single-cell sequencing data. Finally, we constructed a planetary diagnostic view and performed a drug sensitivity analysis to illustrate the clinical contribution of the results.

## Materials and methods

### Data sources

The HM450k DNA methylation data were downloaded from The Cancer Genome Atlas (TCGA, https://portal.gdc.cancer.gov/) database and GSE41826 in Gene Expression Omnibus (GEO, http://www.ncbi.nlm.nih.gov/geo/), which includes 155 tumor samples and 56 normal samples. The methylation level of each probe was represented by the β-value (from 0 to 1). β-Value = I_meth_/I_meth_ + I_unmeth_, I_meth_ is the intensity of methylation, and I_unmeth_ is the intensity of unmethylation. CpG methylation probes were annotated with the platform annotations in GEO (GPL13534). Clinical information and expression data were downloaded from the TCGA database, and the expression level was quantified as fragments per kilobase of transcription per million mapped reads (FPKM) values. Besides, we downloaded gene expression data from the Chinese Glioma Genome Atlas (CGGA, http://www.cgga.org.cn/) database as a supplementary dataset, which includes 282 GBM patients who possessed complete clinical information (Zhao et al., 2021a). The annotation file for mRNAs and promoter region was derived from the GENCODE database (https://www.gencodegenes.org/) (Di Risi et al., 2021). The single-cell sequencing data were obtained from GSE162631 in the GEO database, and cells derived from the tumor cores of three GBM patients in the dataset were selected (Xie et al., 2021a). Expression profile data of human cancer cell lines (CCLs) were obtained from the Broad Institute Cancer Cell Line Encyclopedia (CCLE) project (https://portals.broadinstitute.org/ccle/). Drug sensitivity data of CCLs were achieved from the Cancer Therapeutics Response Portal (CTRPv.2.0, https://portals.broadinstitute.org/ctrp), which contains the sensitivity data for 481 compounds over 835 CCLs (Lauria et al., 2020; Bagaev et al., 2021). The dataset provides the area under the dose-response curve (area under the curve AUC) values as a measure of drug sensitivity, and lower AUC values indicate increased sensitivity to treatment.

### Gene regulation patterns and GBM molecular cluster classification

DEGs were identified with the Limma R package (version 3.48.3), and adjusted *p*-value<0.05 and $\left|\log_{2}Fold\;Change\;\left(FC\right)\right|$ >1 were considered to have a significant difference (Liu et al., 2021). We used the MethylMix R package (version 2.22.0) with the $\left|\log_{2}FC\right|$ >0.5, Cor < -0.3, *p*-value<0.05 to extract the PCGI methylation-driven genes (Xu et al., 2019). Based on the expression of genes, GBM samples were clustered into K (2–9) groups using the ConsensusClusterPlus package (version 1.56.0) in R software (Wilkerson and Hayes, 2010). The optimal K value was determined to obtain a stable cluster, of which correlation coefficients were computed by spearman, and partitioning around medoids was selected as a clustering algorithm.

### Single sample gene set enrichment analysis

The corresponding enrichment score was computed with the GSVA R package (version 1.40.1) (Lauria et al., 2020), which estimated the biological similarity of immune cells by multi-dimensional scaling and a Gaussian fitting model to represent the relative abundance of each immune cell type in gene set enrichment analysis (ssGSEA). Specifically, the tumor microenvironment was assessed by immunohistochemistry for markers of immune cell types (Supplementary Table S1). Further, the ssGSEA score was normalized to unity distribution for each immune cell type, and the estimate scores, including purity, stromal and immune values, were calculated with the estimate R package (version 1.0.13) (Yoshihara et al., 2013).

### Single-cell analysis

We collected three separate tissue samples originating from the tumor core in GBM patients from GSE162631 (21). The raw count data were loaded into the Seurat package (version 4.0.5) for quality control (QC), data filtering, normalization, Principal Component Analysis (PCA), Uniform Manifold Approximation, and Projection (UMAP) visualization, clustering. The single-cell sequencing data from three patients were integrated by the Harmony R package (version 0.1.0) and the cells with mitochondrial genes greater than 10% or fewer than 300 detected genes were filtered out. A scale factor of 10,000 was used to normalize all the remaining cells (Xie et al., 2021a). We used the FindAllMarkers function in Seurat to determine the genes enriched in each cluster and set a logFC threshold of 0.25. It applies a Wilcoxon Rank Sum test and performs multiple test corrections using the Bonferroni method. We used Cellchat R package (version 1.1.3) with the cellchatDB.Human database, which includes supporting evidence for each signaling interaction and considers the structural composition of ligand-receptor interactions and cofactor molecules to identify and visualize cell-cell interactions (Jin et al., 2021).

### Co-expression network

We calculated the Spearman correlation between ligand-receptor genes with PCGI methylation-driven genes. The regulation pairs with Cor>0.4 and *p*-value<0.05 were used to construct the co-expression network, which visualized in Cytoscape (version 3.9.0). We used cytoHubba plug-ins built into Cytoscape to calculate key genes in the network.

### Statistical analysis

All statistical tests were performed in R software (v4.0.3). For the comparisons of the normally distributed groups, statistical analysis was performed by t-tests, and for non-normally distributed variables, statistical analysis was analyzed by Wilcoxon rank-sum tests. The Chi-square test is used to compare clinical, pathological parameters, and other categorical variables. Correlation between two continuous variables was measured by either Pearson’s correlation or Spearman’s correlation. For survival analysis, the differences in prognosis between clusters were assessed via Kaplan-Meier OS analysis, and log-rank tests were utilized to judge the differences between clusters. The cluster prediction model was constructed with LASSO regression in the glmnet R package (version 4.1.2) (Huang et al., 2021). The pROC package (version 1.18.0) in R was utilized to calculate the ROC curves and AUC values. For all statistical analyses, a two-tailed *p* < 0.05 was considered significant. Significance values correspond to *p*-value as follows: ns > 0.05, *<0.05, **<0.01, ***<0.001, ****<0.0001.

### Drug sensitivity

We used the Ridge regression analysis in the pRRophetic R package (version 0.5) to predict differences in drug sensitivity between the two clusters of GBM cancer samples using default settings (Yang et al., 2021). K nearest neighbor imputation was applied to impute the missing AUC values. We used the normalization method to modify the drug sensitivity data matrix of CCLs (Roy et al., 2019). The drugs with $\left|\log_{2}FC\right|$ >0.1 were considered to have differential sensitivity in different clusters (Yang et al., 2021).

## Results

### Identification of glioblastoma clusters based on promoter CpG island methylation-driven genes

To investigate the DNA methylation of promoter CpG island (PCGI) associated with GBM disease progression, we established a richly computational strategy that maps the Infinium HumanMethylation450K microarray to gene PCGI methylation profiles and summarizes DNA methylation patterns at the gene level (Zhao et al., 2021b). First, based on the gene annotation derived from the GENCODE database and GPL13534 platform file containing the methylation probes information (Li et al., 2020; Di Risi et al., 2021), we defined the promoter region as 2 kb located upstream of the transcription start site (Hollstein et al., 2019). We extracted the relevant probes on the PCGI from the annotation file for subsequent analysis (Carro et al., 2010). The mean value for probes was calculated as the methylation level of genes (Liu et al., 2020). In total, 46,072 probes in the DNA methylation microarray were annotated to 15,067 genes, of which we selected 10,895 coding genes according to the gene annotation file. The DNA methylation profiles exhibit the distribution of DNA methylation across the CpG island with a typical DNA hypomethylation tendency in GBM (Supplementary Figure S1A).

**FIGURE 1:**
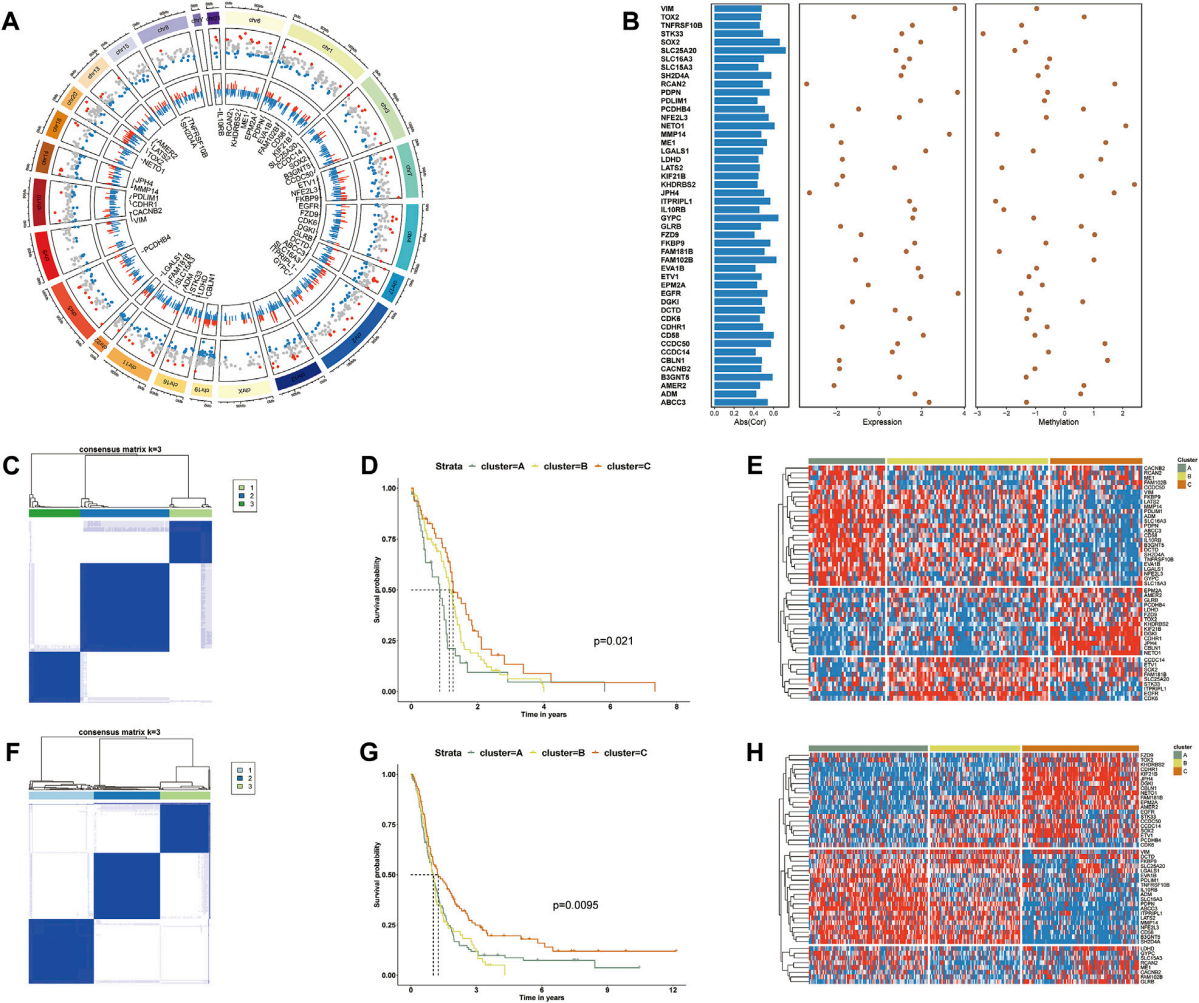
**(A)** The Screening process of PCGI methylation-driven genes. The first circle shows the chromosomal location and ordering information. The second circle represents the distribution of differential methylation genes. Red points represent hypermethylation, and blue ones represent hypomethylation relative to normal samples. The third circle shows the relationship between methylation and gene expression. Blue and red bars represent values with negative and positive correlations, respectively. **(B)** Overview of 48 PCGI methylation-driven genes. The first column shows the absolute values of the correlation coefficients between DNA methylation and expression level. The second and third columns show the fold change of the expression and methylation level. **(C)** Consensus cluster for GBM patients of TCGA based on PCGI methylation-driven genes. **(D)** Kaplan–Meier survival analysis for TCGA sample clusters. **(E)** Hierarchically clustered heatmap for the expression of PCGI methylation-driven genes across clusters in TCGA. **(F)** Consensus cluster for GBM patients of CGGA based on PCGI methylation-driven genes. **(G)** Kaplan–Meier survival analysis for CGGA sample clusters. **(H)** Hierarchically clustered heatmap for the expression of PCGI methylation-driven genes across clusters in CGGA.

Overall, studies on DNA methylation are thought to be associated with an opposite gene expression pattern. Thus, we identified the differentially expressed genes (DEGs) with Limma R package (*p*-value<0.05; $\left|\log_{2}FC\right|$ >1; Supplementary Figure S1B) and calculated the methylation differences and the correlation between expression and methylation with MethylMix R package ( $\left|\log_{2}FC\right|$ >0.5; Cor ≤ -0.3; *p*-value<0.05). Ultimately, we identified 48 PCGI methylation-driven genes (Figures 1A,B and Supplementary Table S2) (Xu et al., 2019).

To clarify the heterogeneity of PCGI methylation-driven genes in TCGA-GBM tumor samples, we performed the consensus cluster method to cluster the samples based on the similarity of PCGI methylation-driven genes expression signature (Datta et al., 2021). It is worth noting that all samples were likely categorized into three clusters named ClusterA, ClusterB, and ClusterC because the interference between clusters can be minimized when K = 3 was selected (Figure 1C and Supplementary Figures S1C–L) (Gong et al., 2020). The epigenomic analysis demonstrates that GBM patients exhibit different levels of abnormal methylation in promoter CpG island, reflecting the heterogeneity of GBM. Particular clustering results for each sample are listed in Supplementary Table S3. The prognostic characteristics of clusters were further appraised by survival analysis, indicating that PCGI methylation is a significant prognostic factor in GBM patients (Figure 1D). The heatmap showed significant disparities in PCGI methylation-driven genes between clusters (Figure 1E). We further collected 283 GBM samples from the Chinese Glioma Genome Atlas (CGGA) RNA‐seq database with clinical information data available and performed the analogous analysis to verify the rationality of results obtained from TCGA(19): we determined the clustering results for CGGA patients based on similarity in gene expression and calculated the survival probabilities between different clusters (Figures 1F,G). Of particular interest, the expression pattern of PCGI methylation-driven genes in the CGGA database is similar to that of the TCGA database, with samples divided into three clusters based on gene expression (Figure 1H). As could be expected, we observed high concordance between the clustering results and prognostic features of CGGA and TCGA.

### Tumor microenvironment heterogeneity between glioblastoma clusters

**FIGURE 2:**
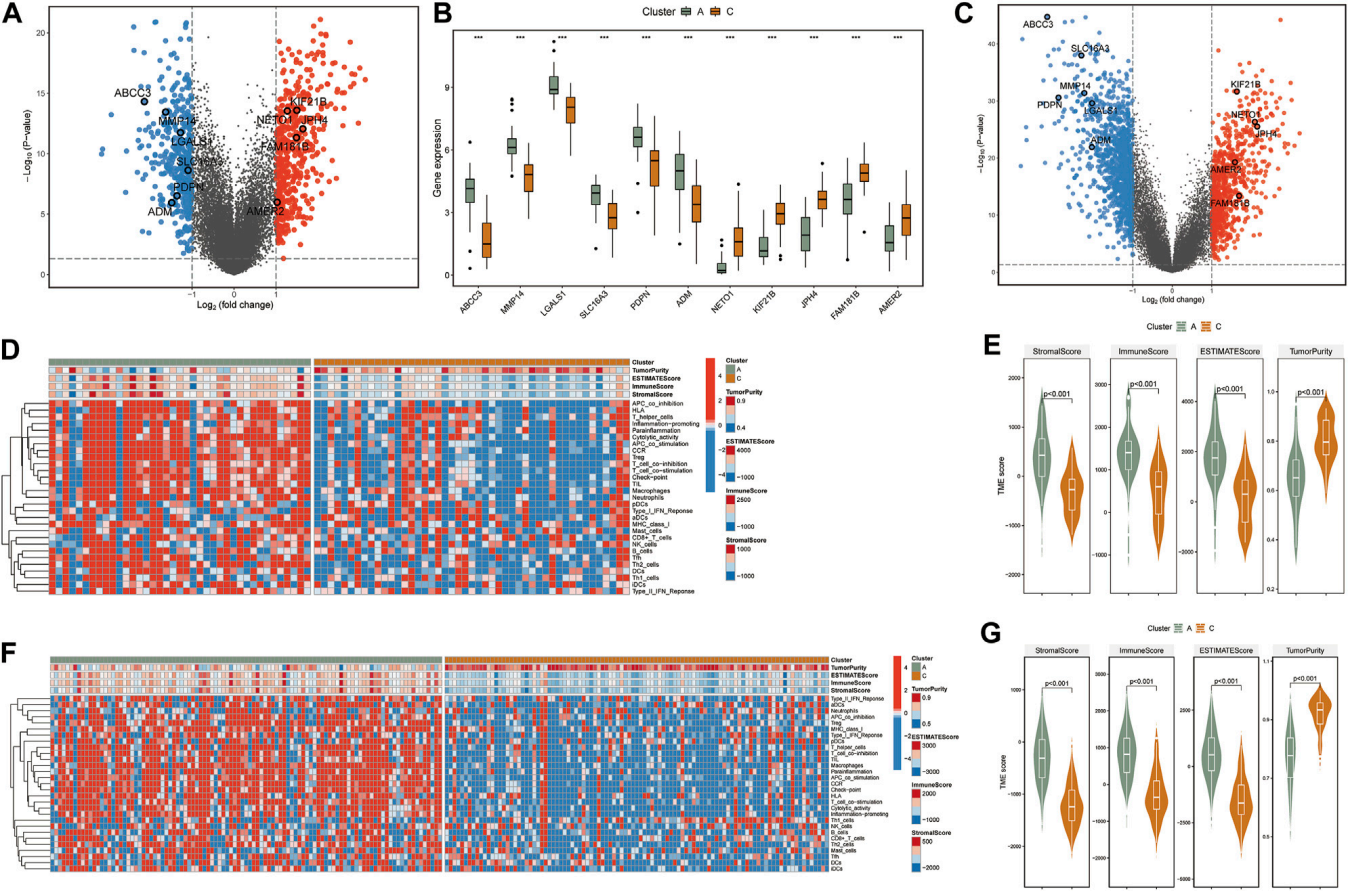
**(A)** The volcano plot shows fold changes for genes differentially expressed between TCGA-clusters A and C. The differentially expressed PCGI methylation-driven genes are highlighted in the figure. **(B)** Boxplots of differentially expressed PCGI methylation-driven genes between clusters A and C. **(C)** The volcano plot shows fold changes for genes differentially expressed between CGGA-clusters A and C. The differentially expressed PCGI methylation-driven genes are highlighted in the figure. **(D)** Heatmap and hierarchical clustering of normalized immune cell infiltration score of TCGA samples. **(E)** Violin plots for the distributions of StromalScore, immuneScore, ESTIMATEScore, and TumorRurity of TCGA samples. **(F)** Heatmap and hierarchical clustering of normalized immune cell infiltration score of CGGA samples. **(G)** Violin plots for the distributions of StromalScore, immuneScore, ESTIMATEScore, and TumorRurity of CGGA samples.

As substantial changes in the tumor microenvironment with infiltrating immune cells and gene regulation machinery can influence tumor progression (Tian et al., 2021), we put the DNA methylation data into a broader GBM context to identify the effect of PCGI methylation in-depth on the tumor microenvironment. We first identified the DEGs between clusters with significant survival differences (clusters A and C) and performed the single sample gene set enrichment analysis (ssGSEA) analysis based on the immune cell signature gene set to investigate the differences between clusters (Bastola et al., 2020; Krug et al., 2020). The results showed that specific PCGI methylation-driven genes were substantially different between clusterA and clusterC within the top differentially expressed genes, including *KIF21B*, *JPH4*, *NET O 1*, *FAM181B*, *AMER2* being up-regulated in clusterC, while *ABCC3*, *MMP14*, *LGALS1*, *SLC16A3*, *PDPN*, *ADM* exhibiting up-regulated in clusterA (Figures 2A,B). Additionally, we got the same results in the CGGA database (Figure 2C). Remarkably, we observed significant differences in the immune cell infiltration between the two clusters. The abundance score of immune cells calculated with ssGSEA was lower in clusterC and higher in clusterA, as shown in Figure 2D. Collectively, it is worth investigating this apparent inconsistency between clusterA and clusterC in the tumor microenvironment as a possible reason for the difference in clinical survival of patients (Gangoso et al., 2021). We further utilized the ESTIMATE R package on the expression profiles of TCGA samples to infer immune and stromal scores for estimating Tumor Purity, Stromal, and Immune Scores (Figure 2E) (Riaz et al., 2017; Stewart et al., 2017; Krug et al., 2020). Studies exist demonstrating that the mesenchymal subtype has many immune cells, in concordance with our work, this subtype showed lower cell density and large necrotic areas in histopathology (Klughammer et al., 2018). We observed high levels of macrophages in clusterA and low levels in clusterC (Figure 2D). We have reviewed the available studies that higher with increased macrophages is associated with lower overall survival (Chen et al., 2019). We also found that the matrix metalloproteinases (MMPs), which might influence the expression of multiple proteins in the extracellular matrix, were differentially expressed between two clusters (Supplementary Table S4) (Theodoris et al., 2015). We speculated that the differences in immune cells could be responsible for the survival status between clusters A and C. Similarly, we obtained practically consistent results by validating with the CGGA database, which proved that our analysis was reliable (Figures 2F,G).

### Linking single cell analysis and communication patterns to glioblastoma clusters

**FIGURE 3:**
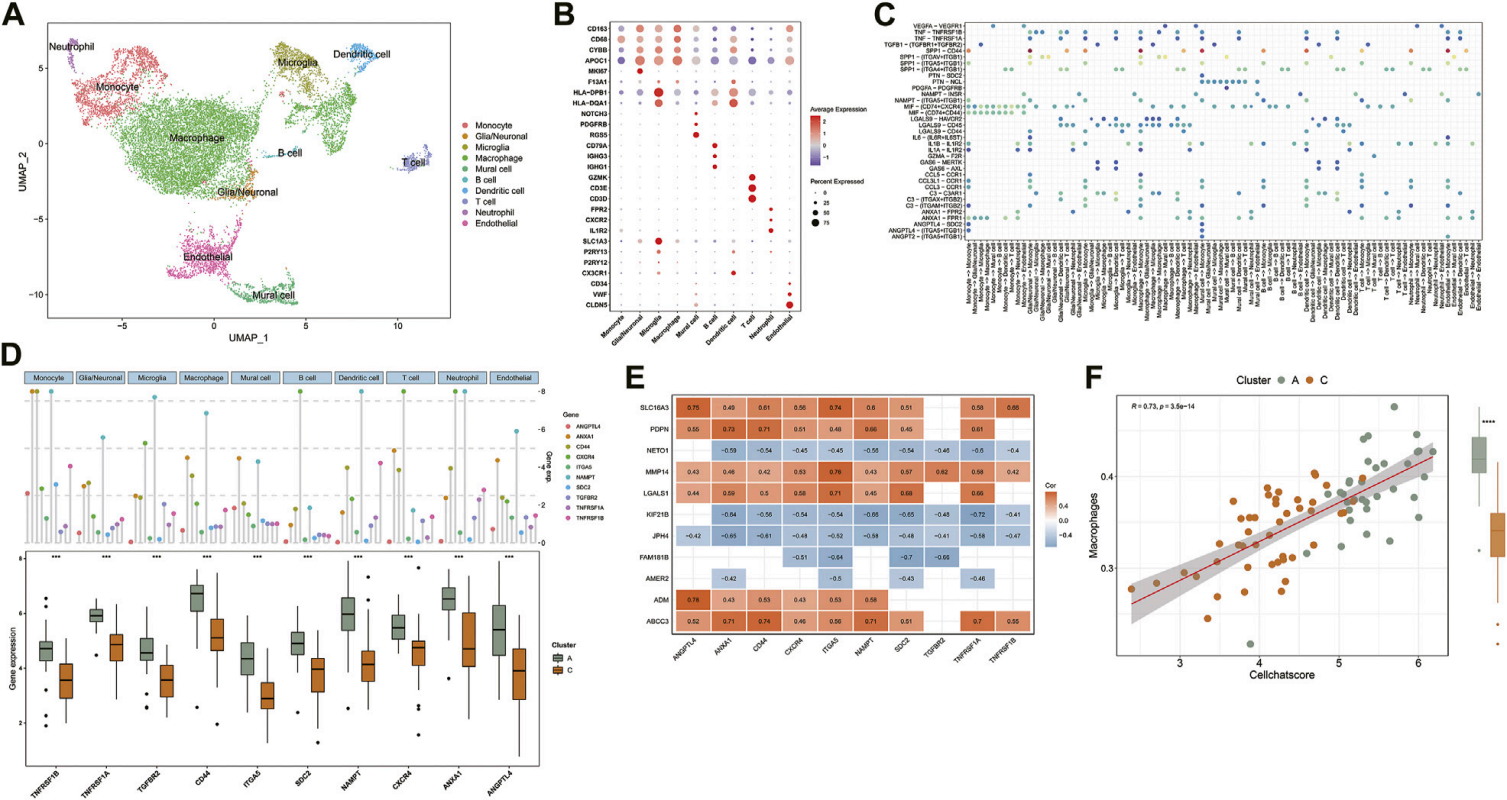
**(A)** UMAP of Single-cell sequencing data, colored for the 10 cell clusters. **(B)** Dot plot heatmap of the marker genes in individual cell clusters. **(C)** The dot plot shows the significant signaling patterns and ligand-receptor pairs. Dot color reflects communication probabilities and dot size represents computed *p*-values. The highlighted signals are pathways in which ligand-receptor genes are differentially expressed between clusters. **(D)** Expression distribution of differentially expressed ligand-receptor genes in 10 cell clusters and comparison of expression between TCGA sample clusters A and C. **(E)** Heatmap shows Spearman’s correlations between PCGI methylation-driven genes and ligand-receptor genes. **(F)** The correlation between Cellchat score and macrophage infiltration score.

To accurately assess the tumor microenvironment between clusters A and C, we analyzed the single-cell data from the core tumor region of three GBM patients (GSE162631). Specifically, the cells were analyzed with the Seurat package in R and annotated according to the expression of canonical cell class markers and the SingleR R package (Xie et al., 2021a; Lu et al., 2021). After the data preprocessing pipeline, the dataset contains 14,926 cells, which cluster into 10 cell groups. The clusters included Macrophages (*APOC1*, *CD163*, *F13A1*), Microglia (*CX3CR1*, *P2RY12*, *P2RY13*), Neutrophils (*IL1R2*, *CXCR2*, *FPR2*), T cells (*CD3D*, *CD3E*, *GZMK*), B cells (*IGHG1*, *IGHG3*, *CD79A*), Dendritic cells (*HLA-DQA1*, *HLA-DPB1*), Glial/Neuronal cells (*FABP7*, *PTPRZ1*), Endothelial cells (*CD34*, *VWF*, *CLDN5*) and Mural cells (*RGS5*, *PDGFRB*, *NOTCH3*) were identified in this data set (Figures 3A,B) (Xie et al., 2021a). We observed a high content of macrophages, monocytes, and microglia in the single-cell sequencing data of GBM samples. Figure 3B illustrates the overlap in gene expression between these 3 cell groups. Consistent with previous studies, the gene expression patterns of these 3 cell groups are similar, and it has always been a challenge to accurately distinguish them in the GBM microenvironment (Ryan et al., 2017; Yao et al., 2020).

Based on published research, we recognized that microglia and tumor-associated macrophages, which accumulate in the tumor region secreting MMPs to promote tumor invasion and secrete tumor cell proliferation promoting factors are distinct subpopulations derived from mononuclear phagocytes (Fan et al., 2020; Ma et al., 2020). The available gene markers do not reliably discriminate between microglia and macrophages. In contrast, the B-cell content was shallow in the GBM microenvironment (Figure 3A). In the central nervous system, B cells are responsible for the antigenic presentation of tumor antigens and participate in anti-tumor immunity (Galstyan et al., 2019).

To predict cell signaling and inferred the precise connections between identified cell clusters to uncover coordinated responses among different cell types. We assessed not only the cell types in the tumor microenvironment but also the interactions between cells within the GBM tumor microenvironment, which constitute an additional layer of information for the integration of DNA methylation data (Lennon et al., 2016).

Normalized single-cell data was then loaded into the Cellchat R package, which integrates cell gene expression and prior knowledge of the interactions between signaling ligands, receptors, and their cofactors to model ligand-receptor mediated signaling interactions (Jin et al., 2021; Leimkuhler et al., 2021). Lastly, we calculated the probability of intercellular communication through Cellchat’s standard process. We detected 35 significant ligand-receptor pairs categorized into 18 signaling pathways, including SPP1, MIF, COMPLEMENT, IL1, ANNEXIN, VISFATIN, GALECTIN, CCL, TNF, PTN, VEGF, GAS, ANGPT, ANGPTL, TGFβ, PARs, IL6, PDGF (Figure 3C). Signaling contribution analysis of cell populations revealed that monocytes were the most important source of SPP1 pathway receptors and the most important source of ANGPTL pathway ligands. Additionally, the communication patterns of multiple cell populations are clustered in the GALECTIN pathway which provided compelling evidence that different cells may depend on the same signals (Supplementary Figures S2D–F).

We further intersected the identified ligand-receptor genes with the list of differentially expressed genes between clusters A and C in the TCGA database simultaneously. The results indicate that ten ligand-receptor genes were differentially expressed between clusters A and C. Notably, all ten ligand-receptor genes were up-regulated in cluster A and down-regulated in cluster C, showing a consistent pattern of differential expression in general (Figure 3D). Compared to the ssGSEA results, these significant differences in the expression distribution trends of the ten ligand-receptor genes in the GBM sample clusters are comparable to the differences in immune cell abundance between clusters A and C (Figures 2D,F). Specifically, multiple signaling pathways may be activated in the tumor microenvironment of subtype A, including TNF, SPP1, MIF, ANGPL, and ANGPTL (Figure 3C). For example, TNF receptor superfamily members might participate in the progression of GBM through responses to TNF signaling pathway and are associated with poor prognosis (Xie et al., 2021b). This cross-referencing of single-cell sequencing data with epigenetic analysis models provides rapid insight into the mechanisms underlying the analysis of the GBM tumor microenvironment.

The correlation between PCGI methylation-driven genes and ligand-receptor genes was further evaluated to explore the effect of PCGI methylation-driven genes on patients’ tumor microenvironment. The correlation heatmap shows that 78.2% of the correlation coefficient matrices had absolute values greater than 0.4, embodying a critical regulatory relationship between PCGI methylation-driven genes and ligand-receptor genes (Figure 3E). Next, we applied weighted co-expression network analysis to the correlation coefficient matrices and explored the critical nodes in the network. Ranked by the degree method, we found that ITGA5 may play an essential role in the network as a key node (Supplementary Figure S2B and Supplementary Table S5). Our results show that the major signaling pathways of ITGA5 include SPP1, ANGPTL, ANGPT, which are characterized by monocytes in the incoming interaction environment, but the communication patterns of outcoming interaction are dominated by macrophages (Supplementary Figure S2C). Then, we defined the mean value of ligand-receptor genes expression in each sample of the TCGA database as a Cellchat score, which quantified the strength of cell communication. We observed that Cellchat score played a significant positive correlation with the abundance of macrophages (Cor = 0.73; *p* < 0.001) (Figure 3F ). Given the crucial role of macrophages in the GBM tumor microenvironment, significant heterogeneity in the expression profile of ligand-receptor genes could help us differentiate the infiltration of macrophages in GBM clusters regulated by PCGI methylation-driven genes, eliminating the dependence of epigenetic typing on high-quality methylation data (Klughammer et al., 2018).

### Building diagnostic map and cluster prediction model for the clinical improvement of GBM clusters

**FIGURE 4:**
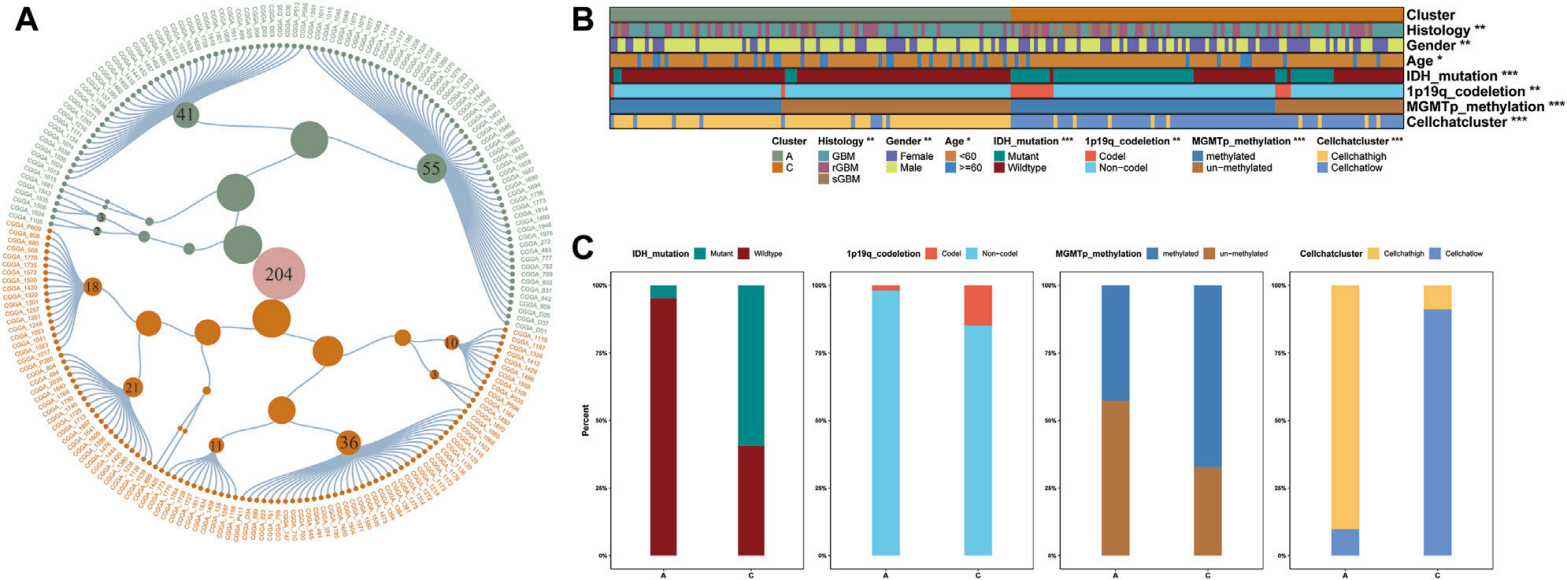
**(A)** Clinical diagnostic map based on GBM samples in CGGA database. Constellation map of GBM sample distribution based on the results of this study. The green points represent clusterA, and orange ones represent clusterC, which is consistent with the color annotation of other figures in this study. **(B–C)** The distribution of CGGA clinical characteristics and Cellchat score we defined.

Precise molecular clustering and clinical features may become key components in prognostic index models (Giacopelli et al., 2019). Therefore, to evaluate the contribution of the GBM clustering results to diagnosis and prognosis in this study, we constructed a diagnostic map containing all clinical features in the database, based on the samples from the CGGA database (Figure 4A and Supplementary Figure S3A) (Bagaev et al., 2021). Additionally, to characterize the clinicopathological relevance of our results, we not only compared the clinical characteristics of the samples in the CGGA database but also calculated the Cellchat score of each sample (Figure 4B). We compared the distribution of multiple clinical features and showed that the majority of patients in clusterA were IDH wild-type. At the same time, we divided the patients into high and low subgroups by the median value of the Cellchat score. The distribution between Cellchat groups and GBM clusters was assessed in the CGGA cohort. The samples in Cellchat high score largely overlapped with clusterA and the Cellchat low score overlapped with clusterC, which showed a comparable outcome to TCGA database analysis (Figure 4C). The reliability of our results was verified in the CGGA database, synthetically validating our conclusions.

**FIGURE 5:**
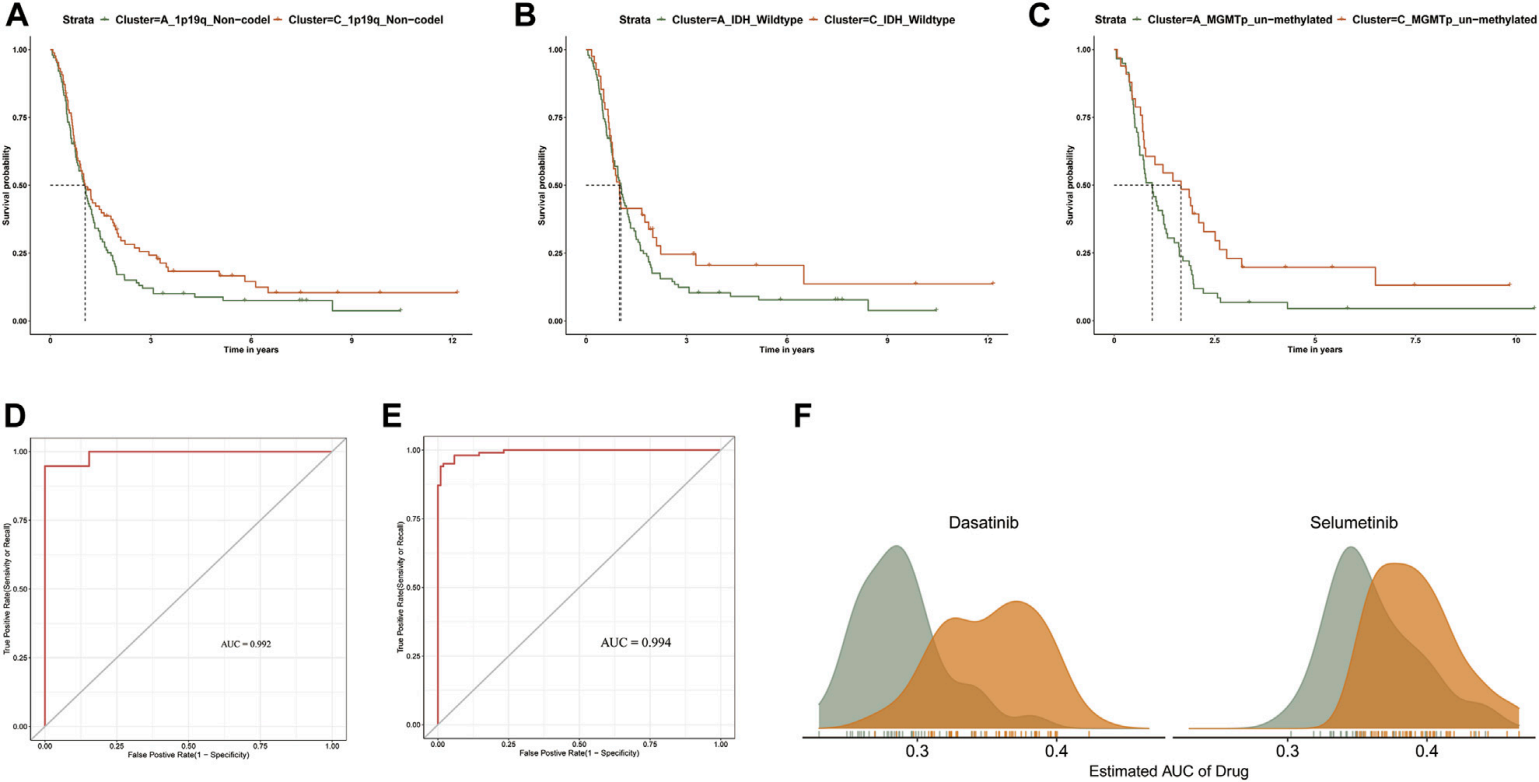
**(A–C)** Kaplan-Meier survival curve analysis between clusters A and C in 1p/19q_Non-codel, MGMTp_un-methylated, and IDH_wildtype). **(D)** The ROC curves of TCGA sample prediction results. **(E)** The ROC curves of CGGA sample prediction results. **(F)** Comparison of estimated Dasatinib and Selumetinib sensitivity.

The clinical utility of our GBM clustering results provided new insights into GBM progression compared to a single clinical feature. For example, we observed that patients with MGMT promoter unmethylated, IDH wild-type, 1p/19q non-codel in clusterC were more likely to achieve a survival advantage at a later stage of GBM progression than patients in clusterA (Figures 5A–C). These findings may suggest that the abnormal methylation profile of promoter CpG islands does not necessarily reflect initial risk factors in GBM progression but is a late result of complex gene-tumor microenvironment interactions throughout GBM progression.

We developed an accurate performance model which can explore a prompt diagnosis (Pyonteck et al., 2013). Specifically, the PCGI methylation-driven genes differentially expressed in clusters A and C were used to construct the prediction model. We randomly divided the samples of TCGA into a training dataset (70%) and test dataset (30%) and brought the CGGA samples as an independent test dataset to verify the repeatability of the cluster prediction model. Then we fit the LASSO logistic regression with the best lambda value to get a stable set of selected features (Supplementary Figures S3B,C). Lastly, the Area Under the Curve (AUC) area was used to quantify response prediction, which exhibited reasonable prediction accuracy in GBM patients with an AUC of 0.975 in the TCGA database and 0.969 in the CGGA database (Figures 5D,E).

For refining the diagnostic map, we concentrated on predicting drug response between GBM clusters based on the CTRP dataset, which contains the gene expression profiles and drug sensitivity profiles of cancer cell lines (CCLs) (Basu et al., 2013; Yang et al., 2021). We excluded the compounds containing NAs in more than 20% of the samples and excluded the CCLs derived from hematopoietic and lymphoid tissue. After pre-processing the data, we used 658 CCLs containing 266 compounds in CTRP and expression profile data from GBM patients to predict patient response to drugs between clusters A and C, based on pRRpphetic with a built-in ridge regression model (Yang et al., 2021). The difference of estimated AUC values of compounds between two clusters was compared with the Wilcoxon rank-sum test, and the results indicated that patients in clusterA showed significantly lower estimated AUC values of Dasatinib and Selumetinib than clusterC (*p* < 0.001) (Figure 5F and Supplementary Figures S3D,E). Previous studies have shown that the combination of Crizotinib and Dasatinib induced an anti-proliferative effect in GBM cell lines, exerting a potent effect on different GBM cell lines when investigating different tyrosine kinase inhibitors (Nehoff et al., 2015; Wang et al., 2020). Additionally, Selumetinib, a kinase inhibitor affecting actionable kinase targets associated with intracranial tumor growth rate, has been selected for single and combination therapy to develop a miniaturized system for drug testing (Gilbert et al., 2018). The difference in estimated AUC values suggest that patients in clusterA may be more sensitive to these two drugs in clinical treatment (Yang et al., 2021). Overall, we believe that our results provide new insights into improving clinical outcomes for GBM patients and the basis for new treatment options for GBM.

### Discussion

Multi-omics data analysis has significantly propelled the understanding of GBM biology, enabling scientists to provide new insights into the GBM precision medicine (Bock et al., 2016). Although the importance of aberrant DNA methylation is well established in various cancers, comprehensive analyses of genomic and single-cell sequencing data based on tumor typing of CpG island within promoter regions remain deficiency. Collectively, elucidating the complexity of the epigenome in GBM typing and therapeutic response specificity may reveal potential mechanisms of targeted therapy and immunotherapy resistance (Manuyakorn et al., 2010). Hence, we performed a consensus clustering analysis with PCGI methylation-driven genes expression profiles and identified three clusters in patients of TCGA and CGGA database, which helped frame the development of GBM precision diagnosis. The identification of PCGI methylation-driven genes comprehensively reflects the influence of methylation information layer on genes and avoids the noise of miscellaneous methylation probe data. Many PCGI methylation-driven genes have been proven extremely valuable in diverse GBM research. For example, the up-regulation of PDPN by cancer cells has recently been linked to an increased risk for venous thromboembolism in GBM (Tawil et al., 2021). Moreover, Hernando et al. found that forced expression of reprogramming transcription factor SOX2, which is highly expressed in GBM, reduces expression of TET2 and 5hmC, thus contributing to the hyper-methylated phenotype of GSCs (Lopez-Bertoni et al., 2022). In terms of other clinical features and diagnostics, our results can complement existing molecular typing while identifying new clinical differences in the integration process.

Because of the particular proliferation form and development process of the tumor, TME exhibits significant differences compared to the normal tissue environment, leading to exclusive characteristics of the tumor [58]. In this study, clusters A and C we identified differed in the degree of immune infiltration in GBM. Combined with the results of single-cell sequencing, differences in the extent of macrophage infiltration in the TME may account for the significant differences in survival between clusters. Macrophages and microglia are significantly abundant in the GBM microenvironment and provide 10%–34% of the tumor mass, which is supported by previous observations (Jacobs et al., 2012). In studies on GBM typing, macrophages and microglia are more increased in recurrent mesenchymal GBM than in primary non-mesenchymal GBM (Wang et al., 2017). Classifying GBM samples based on the TME has predictive power, so efforts to characterize PCGI methylation-driven genes will prove invaluable for identifying the immunosuppressed patients. Additionally, matrix metalloproteinases (MMPs), a key factor degrading almost all proteins in the extracellular matrix, were found substantially distinct between clusters. MMPs can degrade a variety of proteins in the extracellular matrix, and their increased expression levels are positively correlated with the malignancy of GBM. For example, MMP14 was reported to be up-regulated in some types of cancer and to promote cancer cell invasion (Theodoris et al., 2015).

Single-cell heterogeneity, essential for the precise application of biomarkers and selecting appropriate drugs for clinical use, plays an important role in tumor therapy and diagnostic [63]. The signaling pathways identified by the Cellchat R package help us measure the dynamic interactions between tumor cells and their microenvironment. For instance, multiple studies have shown that macrophages maintain GBM cells and stimulate angiogenesis through the SPP1 pathway, which correlates positively with a higher macrophage density in GBM patients. The maintenance of macrophage infiltration and its immunosuppressive phenotype in GBM requires the SPP1 pathway, which induces a positive feedback loop for macrophage production of SPP1 [17]. Previous studies have shown that ITGA5 was increased in GBM tissues and promoted tumor cell proliferation and invasiveness, which is consistent with our results (Figure 3D). Further experiments revealed that NEAT1 promoted ITGA5 expression through competitive binding with miR-128–3p, which might offer a potential strategy for the treatment of GBM (Chen et al., 2021; Shaim et al., 2021). Although many methodological issues need further discussion, the ligand-receptor genes differently expressed between clusters validate the reasonableness of the typing results from different perspectives, indicating the combination of gene methylation and TME may be a beneficial strategy for GBM patients.

Lastly, the diagnostic map refined the former classification and proposed new points for molecular typing [63]. As new criteria and classification methods provide a more detailed understanding of GBM, relying exclusively on a single molecular marker could not satisfy an accurate diagnosis. The observed GBM sample clusters based on PCGI methylation-driven genes in this study improve homogeneous tumor diagnosis and provide insights into the prognosis of GBM patients at later stages of progression (Brennan et al., 2013; Geisenberger et al., 2015). Strikingly, the Cellchat score we defined distinguished the GBM subtypes with clear separation in the CGGA and TCGA databases. This comprehensive DNA methylation- and tumor microenvironment-based classification of biomarker arrays improves molecular understanding of pathway signaling among GBM cell clusters. Here, our results also show that sample classification of GBM can further stratify patient response to different drugs, which could ultimately compensate for personalized therapies in groups of GBM patients.

In conclusion, the results of our analysis adequately discuss the heterogeneous profile of promoter CpG island methylation in GBM. The GBM typing constructed by integrating PCGI methylation-driven genes and the GBM tumor microenvironment in our study contributes to improving the understanding of homogeneous intra-tumor diagnostics.

## Data Availability

The original contributions presented in the study are included in the article/Supplementary Material, further inquiries can be directed to the corresponding author.

## Abbreviations

PCGI, promoter CpG island; MMPs, matrix metalloproteinases.
