# Supplementary figures and images for: Regulatory pattern of abnormal promoter CpG island methylation in the glioblastoma multiforme classification
Source: Front Genet. 2022 Sep 19;13:989985. doi: 10.3389/fgene.2022.989985 (PMC9527345; doi:10.3389/fgene.2022.989985)

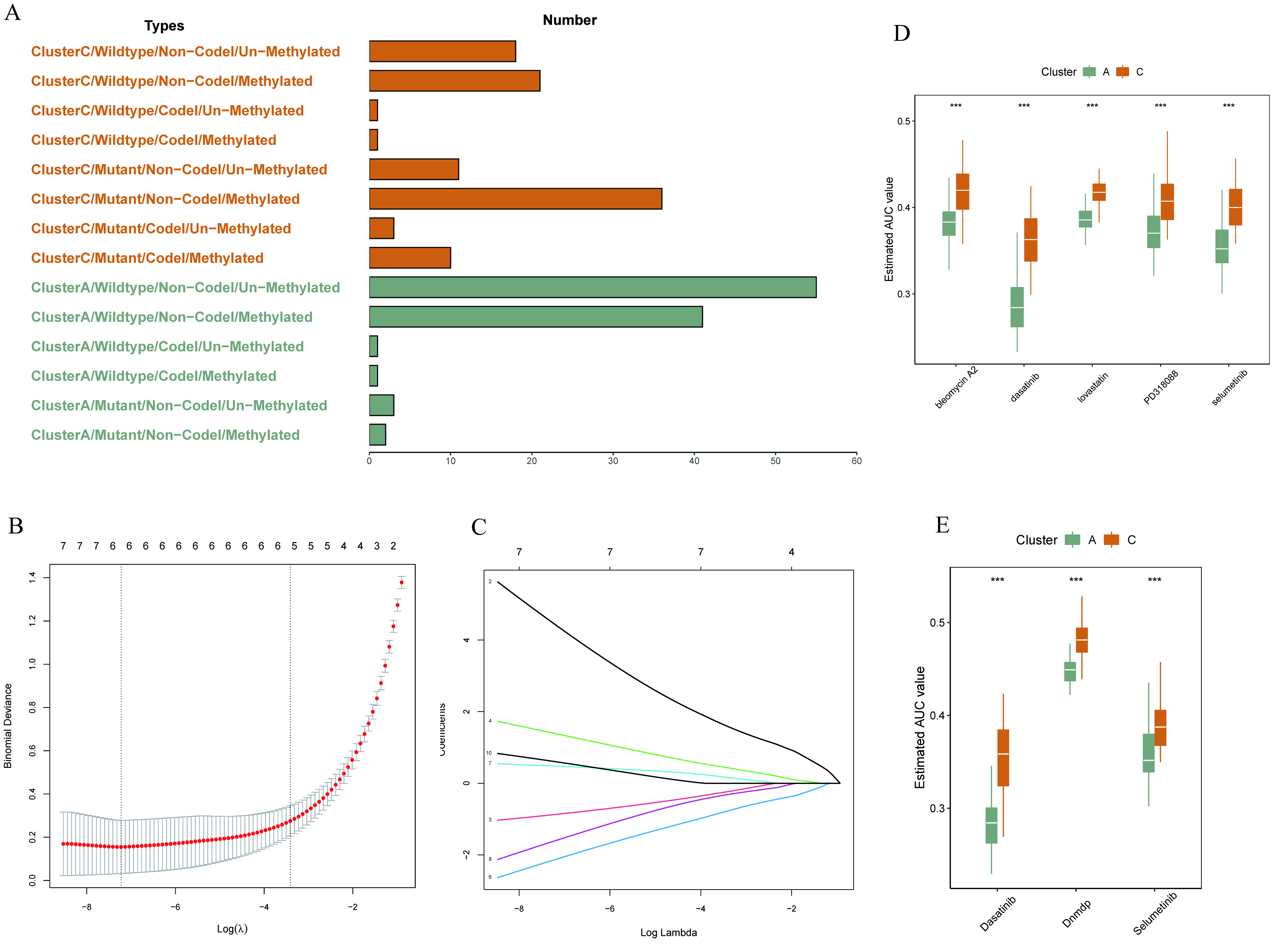

Supplement: Supplementary file 1 [file Image3.TIF]

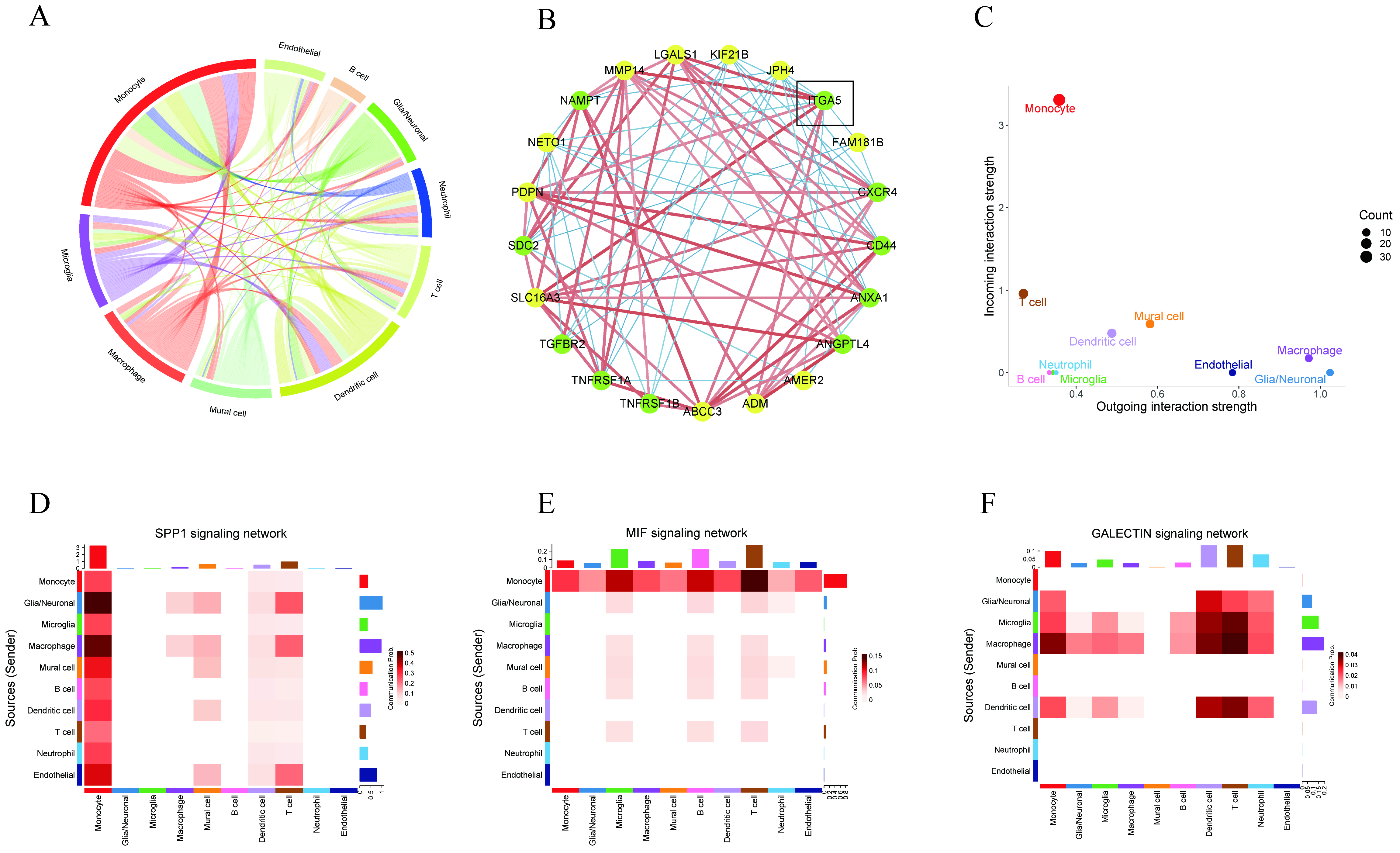

Supplement: Supplementary file 2 [file Image2.TIF]

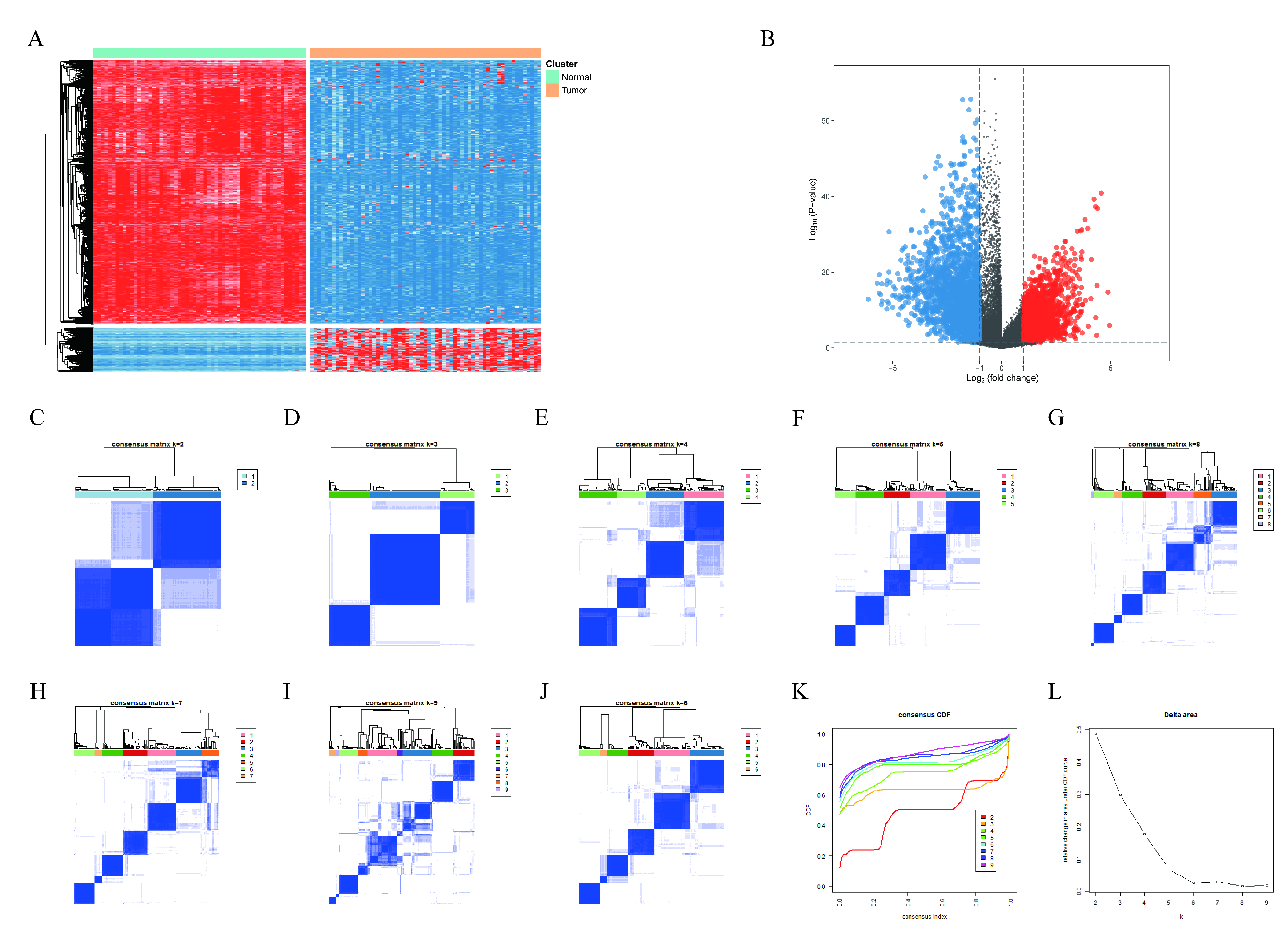

Supplement: Supplementary file 3 [file Image1.TIF]
